# Prevalence and Associations of Depression in Parents of Children With Congenital Talipes Equinovarus: A Single-Centre Study

**DOI:** 10.7759/cureus.61487

**Published:** 2024-06-01

**Authors:** Vikas Verma, Syed Faisal Afaque, Bandana Gupta, Suresh Chand, Durga Narayandas, Udit Agrawal

**Affiliations:** 1 Paediatric Orthopaedics, King George's Medical University (KGMU), Lucknow, IND; 2 Psychiatry, King George's Medical University (KGMU), Lucknow, IND; 3 Paediatric Orthopaedic Surgery, King George's Medical University (KGMU), Lucknow, IND

**Keywords:** ponsetti, dass 21, conjenital talipes equinovarus, clubfoot, depression in parents

## Abstract

Introduction: Congenital talipes equinovarus (CTEV) is a congenital deformity that requires weekly visits to the hospital for manipulation and corrective cast application, followed by an intensive bracing regimen requiring multiple visits to the hospital spread over the years. Parents of children with clubfoot are known to undergo a range of negative emotions. The objective of this study was to identify the prevalence of depression and the factors associated with depression in parents of children with idiopathic CTEV.

Methods: This cross-sectional study consecutively enrolled 190 parents of children with idiopathic CTEV undergoing treatment at King George Medical University. Parents with conditions that preclude the assessment of mental status were not included. These conditions include a history of head injury or psychiatric illness, parents with ongoing treatment of psychiatric illness, ongoing chronic illness, chronic neurological disease, and parents with clinically established intellectual disability. Information was recorded on certain parent-related characteristics and certain child-related characteristics. Parent-related information included age and sex of the parent, religion, area of residence, number of children in the family, degree of perceived social support (using the Multidimensional Scale of Perceived Social Support, MSPSS), level of education, socio-economic status, depression subscale score of DASS 21 (Depression, Depression Anxiety, and Stress Scale -21), chronic pain (visual analogue scale, VAS), family history of clubfoot or depression, and level of stress caused by a major life event during the past year using the Presumptive Stressful Life Event Scale (PSLES). Child-related information included the sex of the child, phase of treatment (casting or bracing), limb involvement (unilateral or bilateral), relapse of the deformity, and Pirani score of the deformity. Bivariate analysis and logistic regression were used to identify factors associated with a score ≥10 on the depression subscale of DASS 21.

Results: One hundred forty-five subjects were males (76.3%). The mean age of the enrolled parents was 28.47±4.89 years. The mean score on the depression subscale of DASS-21 was 4.87±6.3. Thirty-two parents (16.8%) had a score of ≥10 on the depression subscale of the DASS-21. On bivariate analysis, female sex, being Hindu, having studied up to class 12th, relapse, MSPSS score, and PSLES score were found to be associated with a score ≥10 on the depression subscale of the DASS-21. On logistic regression, female sex, lack of graduate education and above, and MSPSS scores were found to be significantly associated with a score of ≥10 on the depression subscale of the DASS 21 score.

Conclusion: The prevalence of depression in parents of children with idiopathic clubfoot was 16.8%. Female gender, lack of college education, and the level of perceived social support (MSPSS) are independently associated with a score ≥10 on the depression subscale of DASS 21. We recommend screening parents of children with clubfoot and referring those with abnormal scores to a psychiatrist for a confirmed diagnosis.

## Introduction

Congenital talipes equinovarus (CTEV), or club foot, is one of the most common orthopaedic congenital deformities [1]. The affected foot or feet appear to be internally rotated at the ankle. The affected foot points downward, and the soles of the feet tend to face each other. The deformity may be unilateral or bilateral, with a higher incidence in males [2]. The gold standard of treatment is the Ponseti technique, which consists of two phases. These include a phase of repeated corrective manipulations and cast applications, followed by a phase of bracing until the child is five years of age. Some children may require a tenotomy of the tendoachillis tendon during the phase of repeated manipulation and cast application.

Depression is one of the more common mood disorders. It is a serious condition that is distinguished by a persistent state of low mood and dislike of activity that can affect an individual’s line of thinking, conduct, affectivity, and perception of well-being [3]. A number of factors are known to be associated with depression. These include age [4], lack of social support [5], female sex [6], family history of depression [6], socioeconomic status [6], chronic disease [7], major life events [7], and substance abuse [8]. Several studies have reported the use of cannabis to be associated with an increased risk of depression [9,10]. Clinical studies have revealed that chronic pain, as a stress state, often induces depression [11].

A child born with a congenital deformity comes as a big shock for the parents. Deformity of the foot becomes a source of worry for the parents as well as the extended family. Increased emotional distress has been reported in parents of children diagnosed with club feet during pregnancy and/or after birth [12]. Parents of children with clubfoot must learn to accept the deformity and undergo weekly hospital visits for casting, followed by an intensive bracing regimen for the child. Parents of children with clubfoot are known to undergo a range of negative emotions like emotional distress, fear, anger, and anxiety [13].

The Depression, Anxiety, and Stress Scale 21 [DASS-21] is a quantitative measure of the three axes of emotional states, namely depression, anxiety, and stress [14]. It is an abridged version of the Depression, Anxiety, and Stress Scale and contains 21 items (seven each for depression, anxiety, and stress). It is not a categorical measure of clinical diagnosis. It can be used as a tool to assess disturbance and, therefore, identify individuals who are at high risk for further problems. The Hindi version of DASS 21 was validated in India by Kumar et al. in the year 2019 [15].

Clubfoot has been reported to be associated with abnormal DASS 21 scores on the subscale of depression in parents of children with clubfoot [16]. Society in India is a mix of rural and urban, but by and large, it is still rural with a large proportion of joint families. Since the social structure of family and society is different in India, we decided to investigate if the same is true for the Indian setup. The treatment of idiopathic CTEV consists of two phases. These include a phase of repeated corrective manipulation and cast application, which is followed by a phase of brace application. Since the mental state of the parent may be affected by the phase of treatment, we decided to investigate the effect of the phase of treatment on the mental state of the parent. We hypothesized that the sex of the child, relapse of the deformity, and whether the deformity is unilateral or bilateral might have an effect on the mental state of the parent and therefore decided to investigate whether they have an effect on the mental state of the parent or not. Keeping in mind the multi-factorial causation of depression, we decided to conduct a study using logistic regression to investigate the factors independently associated with an abnormal DASS-21 (≥10) score on the subscale of depression in parents of children with CTEV.

The objectives of this study were twofold: first, to determine the prevalence of depression among parents of children with idiopathic CTEV, as indicated by a score of ≥10 on the depression subscale of the DASS-21; and second, to identify the demographic, socioeconomic, and clinical factors associated with such depression in this population.

## Materials and methods

This cross-sectional study was conducted in the clubfoot clinic of the Department of Paediatric Orthopaedics, King George Medical University (KGMU), located in the state of Uttar Pradesh, India. The clubfoot clinic at KGMU was started in 2014 and has enrolled about 2000 children until February 2024. The clubfoot clinic is conducted twice a week (Monday and Wednesday) at the outpatient department of KGMU. The Ponseti technique is used to treat children with clubfoot. Plasters are applied at a minimal cost, and braces are provided free of charge to children with clubfoot. The study was approved by the Institutional Ethics Committee of KGMU (Ref. code: 108th ECM IIA/P8).

The sample size was calculated to be 190 using the thumb rule of 10 subjects per variable (events per variable) [17]. The study population was parents of children undergoing treatment by the Ponsetti technique at the clubfoot clinic of KGMU. A representative sample of parents was ensured by consecutively enrolling parents of children with clubfoot, subject to written informed consent. The duration of enrollment was from June 2021 to December 2023. Subjects were interviewed only once, either during the phase of manipulation and casting or during bracing. We excluded parents with conditions that impair their ability to self-report using DASS-21. These included parents with a history of head injury or psychiatric illness, parents with ongoing treatment of psychiatric illness, ongoing chronic illness, chronic neurological disease, and parents with clinically established intellectual disability. We did not include parents of children with syndromic clubfoot, as we wanted to assess the effect of idiopathic CTEV on the mental state of the parents.

The DASS 21 questionnaire was self-reported by the enrolled parents. The rest of the information (for parents as well as children) was collected by the first author. The Pirani score is used to determine the severity and monitor the progress of the deformity in clubfoot. The range is from zero to six, with a higher score indicating a more severe deformity [18]. The severity of the deformity was recorded by the first author using the Pirani score. The information recorded for parents included their age in completed years, sex, religion, area of residence, number of children in the family, level of perceived social support, level of education, socioeconomic status (having a Below Poverty Line card or not), chronic pain (measured on a visual analogue scale, VAS), family history of clubfoot, family history of depression, level of stress due to any major life event during the past year (using the Presumptive Stressful Life Event Scale score), and substance abuse. The VAS is a pain rating scale first used by Hayes and Patterson in 1921 [19]. Parents with alcohol and cannabis use disorders were identified using the DSM-5 criteria for alcohol and cannabis use disorders [20]. The information recorded for the children included the sex of the child, phase of treatment (manipulation and casting or bracing), limb involvement (unilateral or bilateral), Pirani score (in cases of bilateral deformity, the higher score was used), and relapse of the deformity.

Developed by Lovibond and Lovibond, DASS and its shorter version (DASS 21) are commonly used scales to detect depression, anxiety, and stress in adults [21]. Hindi version of DASS 21 has been reported to be reliable (strong internal consistency and construct validity as shown by Chronbach’s alfa values of 0.998, 0.990, and 0.994 for depression, anxiety, and stress domains respectively), and valid (significant correlation with Hospital Anxiety and Depression Scale Hindi version) [15]. We have used the depression subscale of DASS 21. Scoring information for the depression subscale of DASS 21 is shown in Table 1.

**Table 1:** Showing the scoring information for the depression subscale of DASS 21 *DASS 21: Depression Anxiety Stress Scale

Category	Score on the depression subscale of DASS 21*
Normal	0–9
Mild	10–12
Moderate	13–20
Severe	21–27
Extremely severe	28–42

The multidimensional scale of perceived social support (MSPSS) is a self reported score of subjectively assessed social support with a high degree of reliability as well as validity [22-23]. We used MSPSS to measure the magnitude of perceived social support by the parent. The presumptive stressful life event scale (PSLES) is a self-reported scale used to measure stress [24]. It has been developed in India and works well for literate as well as illiterate populations. It has a list of 51 stressful life events, with scores ranging from 20 to 95. We recorded scores for stressful events for a year preceding the date on which the patient was enrolled. In the case of more than one event, the scores were added up to arrive at the final score. The Pirani score was used to determine the severity of each of the components of the clubfoot deformity [18]. The final score was arrived at by adding up the scores of each of the components of the deformity. In cases of bilateral deformity, we used the higher score.

Statistical analysis

Microsoft Excel (Microsoft® Corp., Redmond, WA) was used to record data on a password-protected computer. Categorical data have been summarized using variable frequency tables. Continuous and ordinal data are summarized using measures of central tendency - mean, median, and mode - along with measures of dispersion like the standard deviation. A univariate analysis was done to identify factors at ≥0.25 level of significance. These were used to develop a logistic regression model to identify predictors of a score of ≥10 on the depression subscale of the DASS-21 score.

## Results

One hundred ninety-eight parents met the inclusion criteria. One hundred ninety parents consented to be included in the study. The mean age was 28.47±4.89 years. Males made 23.7% (N=45), and females made 76.3% (N=145) of the recruited parents. The mean score on the depression subscale of DASS-21 was 4.87±6.3. The means of MSPSS and PSLES scores were 5.25±1.24 and 152.20±186.51, respectively. The mean Pirani score was 1.53±1.58 (median 1.00). The mean VAS score for chronic pain was 0.39±0.96. The mean number of children in the family was 1.71±0.81 (median 2). Thirty-two parents (16.8%) had a score of ≥10 on the depression subscale of the DASS-21. Table 2 shows the frequency distribution of categorical parameters among the recruited parents.

**Table 2:** Showing frequency distribution of categorical parameters N: number of subjects

Variable name	Category	N	%
Area of residence	Rural	99	52.1%
	Urban	91	47.9%
Religion	Hindu	151	79.5%
	Muslim	39	20.5%
Level of education	Graduation and above	105	55.3
	Up to class 12^th^	85	44.7
Socioeconomic status - BPL card	Yes	11	5.8%
	No	179	94.2%
Family history of depression	Yes	10	5.3%
	No	180	94.7%
Family history of club foot	Yes	16	8.4%
	No	174	91.6%
Alcohol use disorder	Yes	5	2.6%
	No	185	97.4%
Cannabis use disorder	Yes	6	3.2%
	No	184	96.8%

Of the 190 recruited parents, 95 (50%) were undergoing manipulation and casting, 95 (50%) were undergoing bracing, 75 (39.5%) had unilateral clubfoot, 115 (60.50%) had bilateral clubfoot, and 52 (27.5%) were cases of relapse. Of the 190 recruited parents, 116 (61.1%) had a male child undergoing treatment for clubfoot, and 74 (38.9%) had a female child undergoing treatment for clubfoot.

On bivariate analysis, female sex, being Hindu, having studied up to class 12th, relapse, MSPSS score, and PSLES score were found to be associated with a score ≥10 on the depression subscale of the DASS-2 (Tables 3-4).

**Table 3:** Association between categorical variables and score ≥10 on the depression subscale of DASS-21 score OR: odds ratio, DASS 21: Depression Anxiety Stress Scale **A p-value of <0.05 was considered significant

Exposed variable	DASS-21 score ≥ 10 on the subscale of depression				Unadjusted OR* (95% CI)	p-value**
	Yes (n=32)		No (n=158)			
	N	%	N	%		
Female sex (N=145)	29	90.6%	116	73.4%	3.5 (1.01–12.09)	0.048
Urban area of residence (N=91)	17	53.1%	74	46.8%	0.777 (0.36–1.66)	0.517
Hindu religion (N=151)	26	93.8%	121	76.6%	4.59 (1.05–20.11)	0.043
Education - up to class 12^th^ (N=850)	6	18.8%	79	50.0%	4.33 (1.69–11.10)	0.002
Has a BPL card (N=11)	3	9.4%	8	5.1%	0.52 (0.13–2.06)	0.349
Family history of depression (N=10)	2	6.2%	8	5.1%	0.80 (0.16–3.96)	0.784
Family history of club foot (N=16)	1	3.1%	15	9.5%	3.25 (0.41–25.54)	0.262
Alcohol use disorder (N=5)	2	6.2%	3	1.9%	0.29 (0.05–1.81)	0.186
Cannabis use disorder (N=6)	2	6.2%	4	2.5%	0.39 (0.07–2.22)	0.289
Female sex of the child (N=74)	12	37.5%	62	39.2%	1.08 (0.49–2.36)	0.854
Bracing (N=95)	16	50.0%	79	50.0%	1.00 (0.47–2.14)	1.000
Unilateral clubfoot (75)	13	40.6%	62	39.2%	0.944 (0.44–2.05)	0.884
Relapse (52)	14	43.8%	38	24.1%	0.41 (0.19–0.90)	0.025

**Table 4:** Association between quantitative variables and a score ≥10 on the depression subscale of DASS-21 score** *A p-value of < 0.05 was considered significant **Applied Mann-Whitney U test for significance. DASS 21: Depression Anxiety Stress Scale

	DASS-21 score ≥10 on the subscale of depression				p-value*
	Yes		No		
	Mean	SD	Mean	SD	
Age	29.50	4.43	28.26	4.97	0.192
MSPSS score	3.90	1.14	5.52	1.07	<0.001
Chronic pain (VAS score)	0.34	0.83	0.41	0.98	0.886
PSLES score	256.69	265.29	131.04	159.08	0.003
Pirani score - higher side	1.72	1.65	1.49	1.57	0.408
Number of children in the family	1.73	0.80	1.70	0.82	0.742

Stepwise logistic regression (using variables ≥0.25 significance on the bivariate analysis) showed female sex, education up to class 12th, and MSPSS scores to be significantly associated with a score of ≥10 on the depression subscale of the DASS 21 score (Table 5).

**Table 5:** Results of logistic regression analysis showing association of variables with a score ≥10 on the depression subscale of DASS-21 score B: beta coefficient, SE: standard error, OR: odd’s ratio *p-value of <0.05 was considered significant

Variable name	B	S.E.	p-value*	OR
Female sex	3.746	1.040	0.000	42.338
Hindu religion	0.739	0.893	0.408	2.094
Education up to class 12th	2.727	0.730	0.000	15.280
Relapse	−1.224	0.635	0.054	0.294
MSPSS	1.555	0.313	0.000	4.735
PSLES	−0.001	0.001	0.321	0.999
Constant	−6.844	1.634	0.000	0.001

## Discussion

The results of this study demonstrate the presence of depressive symptoms in parents of children with clubfoot and the factors that are associated with an abnormal score on the depression subscale of DASS-21. This study is the first to identify factors independently associated with a score ≥10 on the depression subscale of DASS-21. As per a report published by the World Health Organization in 2017, the prevalence of depression among Indians is 4.5% [25]. In our study, 32 (16.8%) of the enrolled parents had a score of ≥10 on the depression subscale of the DASS-21 score, which is much higher than the 4.5% reported for the general population of India, which raises the possibility that the presence of clubfoot in children results in depression in their parents. Parents of children suffering from genetic diseases have been reported to suffer from depression [26]. A study that investigated the mental health of parents of children suffering from genetic diseases reported that 18% of the parents suffered from depression, which is about the same as we have reported [26]. The study did not include parents of children with clubfoot, but what may be true of other genetic diseases may be true for clubfoot as well.

On logistic regression, this study found female sex, education up to class 12th (lack of graduate education and above), and a lower MSPSS score to be associated with a score ≥10 on the depression subscale of DASS-21. Of these, the degree of perceived social support is the only modifiable factor. Ours is the first study to use multivariable analysis to identify factors that are independently associated with an abnormal DASS score for depression.

This study has reported that the female gender is significantly associated with a score ≥10 on the depression subscale of DASS-21. Twenty percent (N=29) of the 145 female parents enrolled in our study reported a score ≥10 on the depression subscale of DASS-21, which is considerably higher than the 5.7% lifetime prevalence of depressive disorders for females in India [27]. Mothers of children with congenital deformities have been reported to be more affected psychologically than fathers [28]. In our study, we found that 20% (N=29) of the 145 female parents reported a ≥10 on the depression subscale of DASS-21, which is much higher than the 8.57% (3 of 35 enrolled fathers) reported by the male parents. Association with the female gender may be explained on the grounds of females having a greater propensity for depression and a greater involvement of mothers in the upbringing of the child.

Our study reports a lack of college education (i.e., education up to class 12th) to be significantly associated with a score ≥10 on the depression subscale of DASS-21. Multiple studies have reported the level of education and the presence of depressive symptoms to be inversely related [29-32]. This is in contrast with the results of the study by Fujiwara and Kawachi [33], who did not find any relationship between the level of education and depression. The reasons for the difference could be in the age of the enrolled subjects and in the way the level of education was measured. Fujiwara et al. enrolled middle-aged and older subjects, while the subjects enrolled in our study were young parents. They measured education as a continuous variable (years of education), while we measured it as a categorical variable.

Social support for an individual is broadly understood to be of two types: perceived social support and received social support. Measures to assess perceived social support, like MSPSS, are used to measure an individual’s perception of his family and friends being available to provide material and psychological support. On the other hand, measures to assess social support target specific supportive actions by the support network. A strong relationship between low levels of perceived social support and mental health has been validated [34]. On the other hand, the relationship between mental health and receiving social support is weak [35]. This study has reported a lower MSPSS to be independently associated with a score ≥10 on the depression subscale of DASS-21, which is in keeping with the relationship between perceived social support and mental health [34]. Perceived social support is a modifiable risk factor that is known to improve the quality of life and lower the rates of depressive symptoms [36]. While a psychotherapist who delivers counselling to the parents is employed at certain centres, the results of this study show that there might be a role for counselling in the extended family to improve the level of perceived social support of the parents, which in turn may decrease depressive symptoms in parents.

Stressful life events (SLEs) are known to be associated with depression [37]. We measured stressful life events over the past year using the PSLES score. The mean PSLES score reported by the parents with a score ≥10 on the depression subscale of DASS was significantly higher than that of those with a score <10 on the depression subscale of DASS. However, the significance was lost in logistic regression. A causal relationship between stressful life events and depression has been reported for violent and economically stressful events. PSLES includes stressful life events other than violent and economically stressful life events [37]. This might be the reason for the lack of significance in logistic regression analysis. Perception by an individual about the SLE as being uncontrollable is significantly associated with depression [38]. We did not record any information on the way the parents perceived the stressful life event, which in turn may have resulted in the inclusion of stressful life events about which the parents felt that they were controllable. This could be another reason for the lack of significance that we have reported on logistic regression. This is a limitation of our study. We recommend a study wherein information is collected about specific SLEs known to be associated with depression, their frequency, and the parent’s perception about the SLE in order to delineate the relationship between SLEs and depression in parents of children with clubfoot.

We found relapse of the deformity to be significantly associated with a score ≥10 on the depression subscale of DASS-21 on bivariate analysis. However, the significance was lost in logistic regression. The reason could be that since the parents were already counselled about the possibility of relapse at enrolment, they perceived it as a complication that was treatable, and hence relapse did not have a significant effect on the mental state of the parents.

A shortcoming of this study is that instead of recording information on the religiosity of the parents, we have recorded data on the specific religious affiliations of the parents. A higher degree of religiosity has been reported to be negatively associated with depression [39]. We recorded data on the specific religious affiliation of the parents and found that being Hindu was significantly associated with a score ≥10 on the depression subscale of DASS-21 on bivariate analysis. However, the significance was lost on logistic regression, which is on expected lines, as the degree of religiosity rather than a specific religious affiliation is known to be associated with positive mental health [40].

## Conclusions

The prevalence of depression in parents of children with idiopathic clubfoot was 16.8%. Female gender, lack of college education, and the level of perceived social support (MSPSS) are independently associated with a score ≥10 on the depression subscale of DASS 21. The level of perceived social support is a modifiable factor, and therefore, there may be a role for extending counselling to the family to prevent depressive symptoms in the parents. Since a substantial number of parents of children with clubfoot have depressive symptoms, we recommend screening parents of children with clubfoot, and those with abnormal scores should be referred to a psychiatrist for a confirmed diagnosis.
